# Sustainable food systems—a health perspective

**DOI:** 10.1007/s11625-018-0586-x

**Published:** 2018-06-12

**Authors:** Elisabet Lindgren, Francesca Harris, Alan D. Dangour, Alexandros Gasparatos, Michikazu Hiramatsu, Firouzeh Javadi, Brent Loken, Takahiro Murakami, Pauline Scheelbeek, Andy Haines

**Affiliations:** 1 Stockholm Resilience Centre, Stockholm University, Kräftriket 2B, SE-106 91 Stockholm, Sweden; 2 Swedish Institute for Global Health Transformation, Royal Swedish Academy of Sciences, Stockholm, Sweden; 3 Department of Population Health, London School of Hygiene & Tropical Medicine, London, UK; 4 Integrated Research System for Sustainability Science (IR3S), University of Tokyo, Tokyo, Japan; 5 Institute of Decision Science for a Sustainable Society, Kyushu University, Fukuoka, Japan; 6 EAT Foundation, Stockholm Resilience Centre, Stockholm, Sweden; 7 Departments of Population Health and of Public Health, Environments and Society, London School of Hygiene & Tropical Medicine, London, UK

**Keywords:** Sustainable diets, Food waste, Industrial crops, Food security, Sustainable Development Goals SDGs, Sustainable food systems

## Abstract

Malnutrition in all forms, ranging from undernourishment to obesity and associated diet-related diseases, is one of the leading causes of death worldwide, while food systems often have major environmental impacts. Rapid global population growth and increases in demands for food and changes in dietary habits create challenges to provide universal access to healthy food without creating negative environmental, economic, and social impacts. This article discusses opportunities for and challenges to sustainable food systems from a human health perspective by making the case for avoiding the transition to unhealthy less sustainable diets (using India as an exemplar), reducing food waste by changing consumer behaviour (with examples from Japan), and using innovations and new technologies to reduce the environmental impact of healthy food production. The article touches upon two of the challenges to achieving healthy sustainable diets for a global population, i.e., reduction on the yield and nutritional quality of crops (in particular vegetables and fruits) due to climate change; and trade-offs between food production and industrial crops. There is an urgent need to develop and implement policies and practices that provide universal access to healthy food choices for a growing world population, whilst reducing the environmental footprint of the global food system.

## Introduction

Our world is rapidly changing. Increasing food consumption by a growing population, together with changing dietary habits, pose an immense challenge for the global food system. A crucial question is how to meet the increasing demand for food and provide healthy diets for all for the decades to come without undermining the Earth’s resources and crossing planetary boundaries, beyond which the future prospects for humanity may be threatened (Godfray et al. 2010; Steffen et al. 2015).

The world’s population has increased by two billion during the last 25 years, and is projected to reach 8.5 billion by 2030 and 9.8 billion in 2050 (United Nations World Population Division 2017). The Millennium Development Goals contributed to a fall in the percentage of undernourished people from 23.3% in 1990–1992 to 12.9% in 2014–2016, but there remain more than 800 million undernourished persons in the world and the absolute numbers are increasing (FAO et al. 2017, UN 2015a). The Sustainable Development Goals (SDGs) have the ambition not only to end poverty and hunger by 2030, but also to ensure that “all people, at all times, have physical, social and economic access to sufficient, safe and nutritious food which meets their dietary needs and food preferences for an active and healthy life” (UN 2015b). This puts a focus on all aspects of malnutrition and the food system.

Nearly, one-third of the global population is currently suffering from some form of malnutrition; ranging from undernourishment (i.e., the main hunger indicator), stunting (short-for-age), micronutrient deficiencies, to overweight and obesity with associated risk of non-communicable diseases (NCDs), e.g., diabetes, cardiovascular diseases, and cancer. The prevalence of obesity is increasing and currently affects about 13% of the adult global population (FAO et al. 2017). The double burden of malnutrition refers to the coexistence of obesity and undernutrition as a consequence of high energy diets with poor nutritional content, and is a condition of increasing concern in areas with rapid nutrition transitions.

Between 2001 and 2011, the global middle class (depending on how middle class is defined in different countries) doubled to at least 13% of global population with most of the growth occurring in Asia and the South Pacific, where now half of the world’s middle class is living (Pew 2017). A global dietary study of 187 countries found that a dietary shift towards unhealthy food items (i.e., red meat, processed meat, and diets high in saturated fat, trans fat, free sugar, and sodium) also occurred in large parts of Asia between 1990 and 2010 (Imamura et al. 2015). This indicates that the transitions from low income predominantly rural populations, towards middle income and urban populations, particularly in emerging economies, is often associated with changing dietary patterns (Satterthwaite et al. 2010). The transitions from traditional diets towards diets that include more unhealthy food items will increase the risk of diet-related NCDs (Iso 2008).

Food systems^1^ can have major environmental impacts. Food production may, for example, have impacts on land degradation, deforestation, loss of habitats and biodiversity, depletion of natural resources, and contamination of air, soil and waters (IPBES 2018; Whitmee et al. 2015). For example, food systems account for about one-fourth of anthropogenic greenhouse gas (GHG) emissions, and agricultural production for 70% of global fresh water withdrawals (FAO et al. 2017; Vermeulen et al. 2012). The use of synthetic fertilizers and pesticides in agriculture, and the use of hormones in animal husbandry cause chemical pollution of marine and terrestrial ecosystems with contamination of food products and ecosystems that in turn may lead to severe health consequences (Corsolini et al. 2005; Landrigan et al. 2017; UN Water 2016). Preventive antibiotic use in animal husbandry contributes to antibiotic resistance (Tang et al. 2017; WHO 2017). These practices will, in combination with the growing competition for land, water and energy affect the capacity for future sustainable healthy food production (Garnett 2014; Godfray et al. 2010; Whitmee et al. 2015).

Despite the substantial contribution that the promotion of healthy diets can have for enhancing sustainability, so far, it has seldom been examined within the field of sustainability science, as health is often a peripheral topic (Kajikawa et al. 2014, 2017). This article discusses some of the key interlinkages between healthy diets and sustainable food systems, and covers both opportunities for and challenges to a shift towards healthy sustainable diets. The article touches on issues closely aligned to sustainable food systems such as food waste reduction, and interactions between food and industrial crop systems, and discusses dietary transitions and emerging innovations in an interlinked health and sustainability perspective.

## Interactions between health, food systems, society and the environment

The type of food an individual consumes is influenced by several factors. Affordability and availability are crucial factors (e.g. Miller et al. 2017). However, cultural and religious aspects, personal preferences and health concerns, information, consumer awareness and influences from marketing strategies and trends are also of importance. These determinants of food choice are context specific and different factors dominate within different socio-economic, cultural and geographic settings in low-, middle-, and high-income countries.

The contents of a healthy diet will vary depending on an individual’s nutritional status and dietary needs (e.g., age, gender, health and lifestyle), and the cultural and socio-economic context (WHO 2015). The basic recommendations for a healthy diet for adults are shown in Table 1. The dietary guidelines for Americans (HHS and USDA 2105) recommend that the calorie intake of younger children aged 1–3 compared to older children should consist of more fat (30–40% compared to 25–35%), slightly less protein (15–20% compared to 10–30%) and the same calorie percentage (45–65%) of carbohydrate. Diets rich in dietary fibre, vegetables, fruits, nuts, seeds and unsaturated fats and with a low content of meat, in particular red meat, are associated with reduced risk of heart disease, stroke, type-2 diabetes, and cancer (Boeing et al. 2012; Fung et al. 2002; IARC 2003; Pan et al. 2012; Pereira et al. 2004; Tilman and Clark 2014; Willett 2006).

**Table 1:** Recommendation for a healthy diet for adults (WHO 2015)

Healthy diets for adults	
•	The basic diet should consist of fruits, vegetables, legumes (e.g., lentils, beans), nuts and whole grains, with a daily intake of 400 g of fruits and vegetables
•	Free sugars should be < 10% and fats < 30% of total energy intake
•	Unsaturated fats (e.g., found in fish, avocado, nuts, sunflower, canola and olive oils) are preferable to saturated fats. Industrial trans fats, found in processed food should be avoided
•	The intake of salt should be < 5 g per day

Sustainable diets are not only nutritionally beneficial, but also consider wider aspects of global sustainability (Meybeck and Gitz 2017). The Food and Agriculture Organization of the United Nations defined sustainable diets as “diets with low environmental impacts which contribute to food and nutrition security and to healthy life for present and future generations. Sustainable diets are protective and respectful of biodiversity and ecosystems, culturally acceptable, accessible, economically fair and affordable; nutritionally adequate, safe and healthy; while optimizing natural and human resources” (FAO 2010, page 7).

**Fig. 1:**
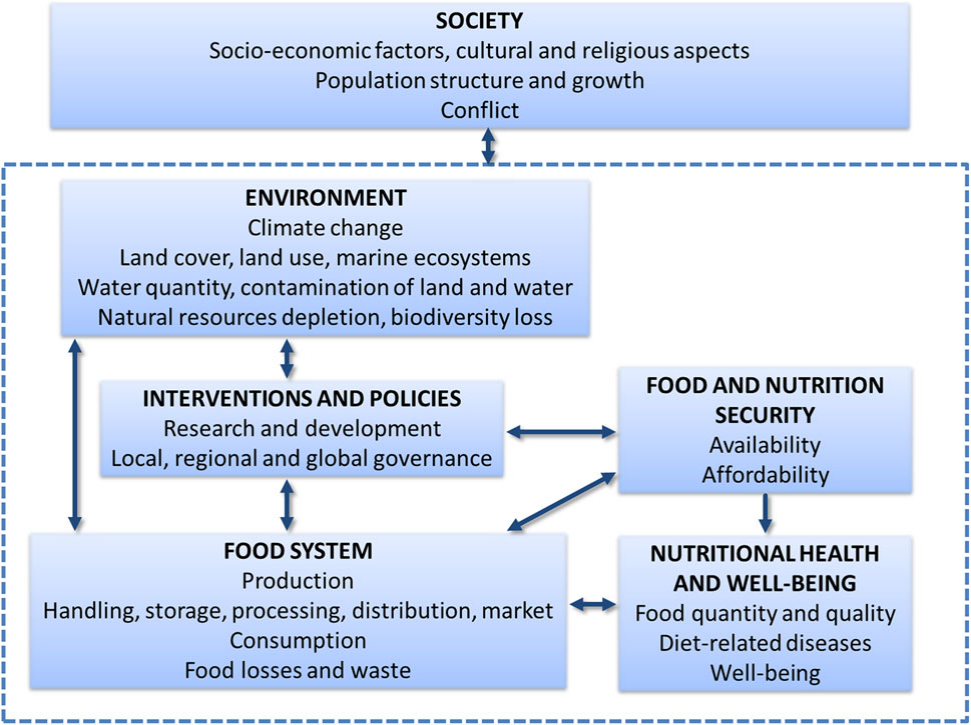
Interactions between health, food systems, environment, and society. Modified from Tuomisto et al. 2017

Figure 1 illustrates interactions between health and well-being, food systems, environmental factors and societal and socio-economic factors. There is a range of changes that can be made within the food system to minimise the environmental burden, ensure economic profitability and equity, and improve health; the latter, for example through reducing undernourishment and exposures to toxic compounds. It is not within the scope of this article to discuss sustainable food systems^2^ and food production practices more in detail. This has been done extensively elsewhere, for example by CGIAR 2018, Foley et al. 2011, Garnett et al. 2013; Rockström et al. 2017; Tilman et al. 2011; Whitmee et al. 2015. However, this article provides examples, viewed from a health perspective, of opportunities of dietary transitions, emerging food innovations and technologies, and initiatives to reduce food waste through behaviour change, but also of challenges to achieve healthy and sustainable diets, as discussed below.

## Opportunities for healthy and sustainable food systems

### Positive dietary shifts

Growing evidence suggest that population-level dietary changes could improve both health and environmental sustainability (Aleksandrowicz et al. 2016; Nelson et al. 2016; Perignon et al. 2017). Thus, a transition to diets that follow consensus national dietary guidelines could substantially reduce the environmental impacts of food systems and improve population health outcomes (Behrens et al. 2017). Animal source foods are high in saturated fats and usually incur higher environmental burdens than plant-based diets, mainly due to larger need of land and to the energy and material inputs required for feed and methane produced from enteric fermentation; the latter specifically of ruminants (Herrero et al. 2013). Thus, switching to healthy plant-based diets with lower animal source food content could reduce GHG emissions and free land, whilst also having co-benefits for health, in particular reductions in non-communicable disease risk (Tilman et al. 2011).

It has been estimated that switching to affordable healthy diets in the UK could reduce the GHG emissions of diets by 17% and at the same time save almost 7 million years of life lost prematurely due to diet-related non-communicable diseases over the next 30 years largely as a result of increased fruit and vegetable consumption (Green et al. 2015; Milner et al. 2015). It should be noted that most research on sustainable diets has mainly quantified environmental impacts in terms of GHG emissions, with some studies also including impacts related to water and land use. However, the published research has focused largely on high-income countries and its applicability to other settings is unclear (Aleksandrowicz et al. 2016). Generalising research results on sustainable diets can be complicated due to the substantial heterogeneity in existing dietary patterns globally, as well as in balancing potential tensions and trade-offs between health, environmental and social constraints and desirable outcomes (Meybeck and Gitz, 2017). However, as diets in rapidly developing countries are typically becoming more resource intensive (Gill et al. 2015), understanding the environmental and health impacts of such dietary transition offers an important opportunity to promote the sustainability of the food systems.

India is such an example. It is a predominantly lacto-ovo-vegetarian society, characterised by large regional differences in diets; populations in the south typically consumed rice as the staple food, while those in the north consume wheat (Joy et al. 2017). However, dietary patterns are changing; intake of calories, fat, sugar and salt is increasing, which is leading to a rapid rise in non-communicable diseases (Misra et al. 2017). Water security is emerging as a major concern for India’s agricultural sector as the use of ground and surface water for food production is comparatively high (Harris et al. 2017). The substantial expected growth in the Indian population up to 2050 suggests that water use for Indian agriculture may increase unsustainably. In this context, recent optimisation modelling has identified that relatively small healthy dietary shifts in India could reduce dietary water footprints to meet future constraints, whilst minimising changes to cost and simultaneously cutting GHG emissions (Milner et al. 2017). If adopted, these diets could improve population health in India; with the potential to gain 6800 life-years per 100,000 total population by 2050 (Milner et al. 2017). However, these dietary changes may need to be more severe in light of additional challenges such as climate change and rapid urbanisation.

More local-level evidence is needed that integrates an understanding of the environmental impacts of specific dietary preferences along with other societal changes. This is particularly true for low- and middle-income countries. It is important for studies that explore the possible effects of dietary shifts to consider simultaneously the impacts of multiple environmental stressors on agriculture.

### Emerging innovations and technologies

The global urban population is rapidly growing. There are growing interests in food production within cities, even though many cities have restrictions for urban farming. There is a range of emerging farming techniques with low environmental impacts that may be used in controlled indoor settings also in cities. Promising are, for example, hydroponic (plants grown in nutrient-rich solutions), aquaponic (using water and fish waste), and aeroponic (nutrient-rich water is sprayed onto—in air—dangling roots) techniques. For example, hydroponic greenhouses on the rooftops of some buildings in New York and Chicago now produce fresh greens with shorter transportation routes and storage time for the local markets (Clinton et al. 2017).

To meet the increasing demand for sustainable healthy food, the food industry is on the lookout for new food items and technologies. Novel food, which means traditional food used for new markets, is also of interest. Edible insects are since long part of the diet in many countries around the world. Since insects are high in protein, with low risks for transmission of zoonotic diseases and have low environmental impacts they are considered novel food products of interests, both from a sustainability and health perspective. Insects can be used either for direct consumption, or indirectly in recomposed foods with protein extracted from the insects (Belluco et al. 2017). Cultivation of aquatic plants (seaweed) is another traditional sector that is rapidly growing and is now practised in about 50 countries together with algaculture for human consumption (FAO 2016, Well et al. 2017).

Emerging innovations and technologies are producing new food items with low environmental impacts. Mycoproteins, for example, are protein substitutes produced from fungal biomass. Cellular agriculture is an emerging industry that focuses on in vitro production of food items such as meat, milk, eggs, and gelatine using animal and plant cells and microorganisms. Cultured meat production emits less GHG and uses less land than conventional red meat production, which increase the area of usable land for other purposes, such as industrial crop production (Tuomisto et al. 2014). Even though promising, more research is needed on the advantages and disadvantages of emerging new food items and their contribution to healthy sustainable food systems.

### Food losses and waste

Approximately, one-third of the food produced for human consumption is lost or wasted every year, which is four times as much food needed annually for eliminating global hunger (FAO 2013). Food losses and waste (FLW) impact the sustainability of food systems across the three dimensions: economic, social and environmental. Food losses and waste have negative economic effects, impede development, hinder social progress, contribute to unnecessary emissions, and undermine food security, but also waste valuable nutrients, energy and natural resources (HLPE 2014; Gustavsson et al. 2011). Reducing FLW may, thus, present a great opportunity in enhancing the sustainability of the food system and simultaneously improve food security and nutrition.

**Fig. 2:**
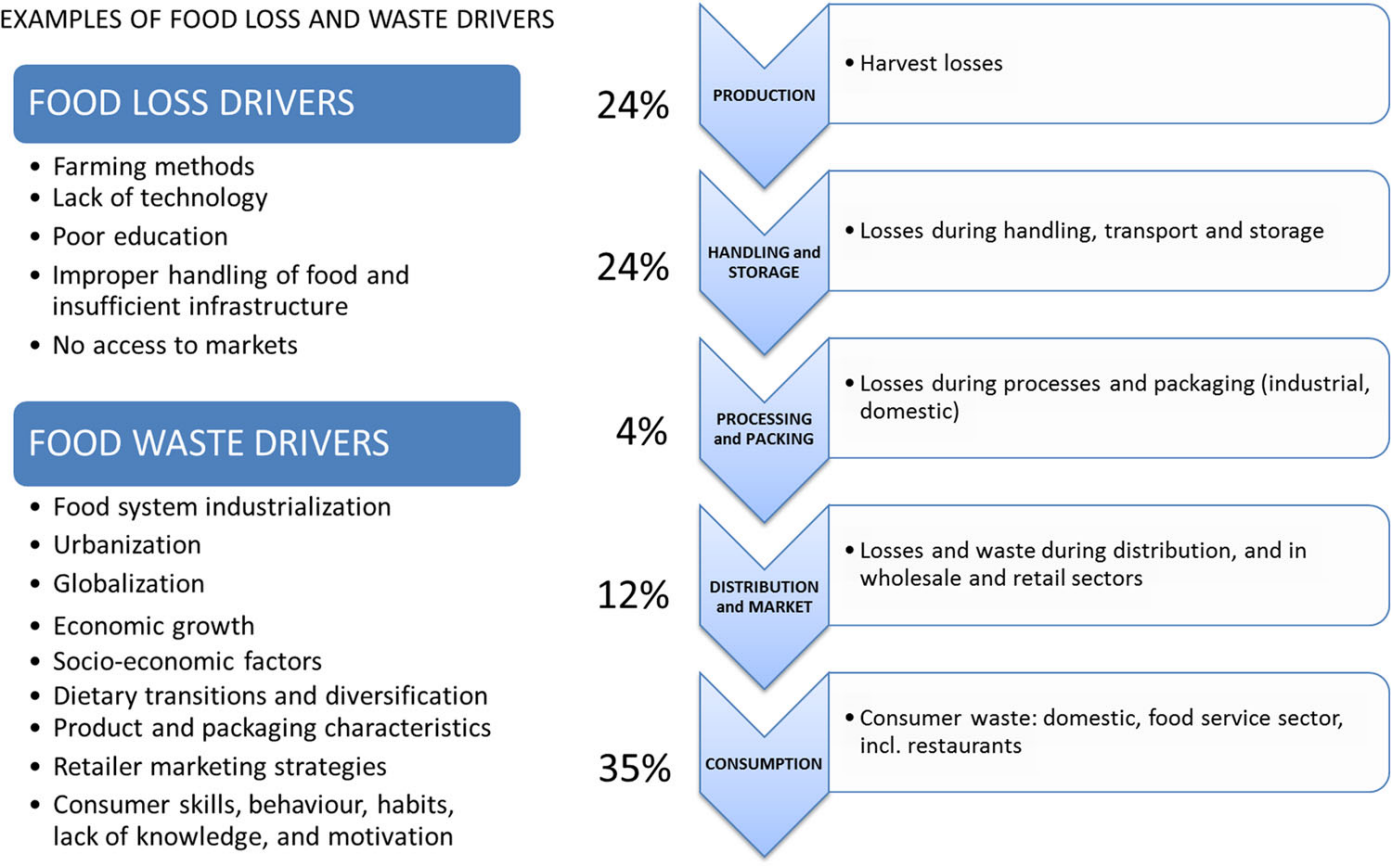
Food losses and waste along the food supply chain, with percentages of total loss/waste in calories (Gustavsson et al. 2011)

Figure 2 outlines the differences between FLW within the food supply chain. Food losses normally occurs in the first stages of the food supply chain and include food that gets spilled or spoilt before it reaches its final product or retail stage (e.g., Corrado et al. 2015; EIU 2014). Food waste refers to food that is fit for consumption but left to spoil or discarded by consumers and retailers. Of all food lost or wasted nearly half, in terms of calories, occur in the first parts of the supply chain, and 35% in relation to consumption (e.g., Aschemann-Witzel 2016; Thyberg and Tonjes 2016; Watabe et al. 2016). However, patterns of FLW are context-specific.

Food losses, in low- and middle-income countries predominantly occur due to inefficient harvesting, insufficient storage facilities and lack of infrastructural conditions and access to markets (EIU 2014). In such national contexts, the solutions for reducing food loss, as well as food insecurity, are often directly related. Three major aspects of the food supply chain must be improved at national and regional levels to reduce food losses: (1) farming methods (e.g., increased mechanization), (2) structural infrastructure (e.g., transport and storage systems and processing facilities), and (3) the operating environment (e.g., facilitate access to markets and efficient markets) (EIU 2014).

The amount of food wasted depend on cultural habits, attitudes, and socio-demographic factors, and on factors such as the consumer’s food storage possibilities, meal planning, shopping behaviour, cooking skills, knowledge of date labelling, and time constraints, among others (Stancu et al. 2016; WRAP (Waste and Resources Action Programme) 2009). Urbanization and dietary transitions in low- and middle-income countries are expected to contribute to increased food waste at the consumption stage in households and catering (Parfitt et al. 2010). In high-income countries, the wasteful practices of the food industry and consumers are the predominant drivers of food waste. For example, it has been argued that reducing consumer food waste would have significant impacts in countries such as the USA, where consumer waste is high. Food waste solutions can be categorized into three levels: (1) reducing waste at the source (i.e., waste prevention); (2) recovery, (e.g., through food donations and redistribution); and (3) recycling, (e.g., waste used for animal feed, energy production, and compost) (Mourad 2016). Food waste prevention requires changes in people’s behaviour, both collectively (e.g., food industry), and individually (BioIntelligence Service 2011).

It has been argued that consumer education can play an important role in reducing food waste, offering an opportunity to enhance the sustainability of the food system. Schools offer a unique opportunity to foster food waste prevention behaviours. Such an example is “Bento Day”, a dietary education program in Japan, that has grown to more than 2000 elementary and junior high schools since 2001. In this educational program, the students cook and prepare at home a “Bento” (i.e., a Japanese single meal box), to bring to school for lunch. Such self-management skills not only help students to better understand the process of cooking, but also contribute to reduce both food waste at home and school lunch leftovers since the children understand the work needed behind food preparation (Takeshita 2006). Such consumer knowledge gained through the children’s cooking experiences may both influence positive consumer behaviour and contribute to food waste reduction.

Social education on “reduce, reuse, recycle” of food waste may contribute to increased sustainable consumption behaviours. There are several options for closing the food waste loop through waste recycling; for example as feed, compost, and fertilizer (Fiala et al. 2016; Salemdeeb et al. 2016; Salomone et al. 2016). Takata et al. (2012) found that animal feed facilities in Japan that recycled food waste as feed were more economically effective than facilities that were not using such recycling food waste loops. If all farmers in Japan were to introduce recycled food waste as pig feed, the economic benefits of food waste reduction would be about 167 million US dollar, while food waste incineration costs 1900 million US dollar (Yu 2008).

Finally, education is valuable in spreading environmental awareness as a means of influencing behaviour toward better food waste management and reduction (Holland et al. 2006; Wu et al. 2013; Young et al. 2013). For example, a research team from Hokkaido University of Education and Ohno Agriculture School in Japan conducted experiments on the lactic acid fermentation of food waste including by-products and leftovers for pig feed. An environmental awareness survey among the participating students indicates that environment-related knowledge and awareness significantly influenced attitudes towards recycling behaviour and a better understanding of sustainable food systems (Wataya and Murakami 2017). A UK study found that environmental awareness could predict an individual’s waste reduction and reuse behaviour whereas recycling was considered as normative behaviour (Bar 2007).

### Economic and fiscal considerations

In addition to information and education of producers, suppliers and consumers, economic and fiscal incentives, including ecotaxes, incentive payments/subsidies, ecolabeling, and environmental markets could be used to promote sustainable agriculture and new food items; lower the environmental impacts of the food processing, transport, storage and retail sectors; and increase food waste reuse and recycling (Sands 2003; Whitmee et al. 2015). Subsidies and taxes are also important for changing consumer behaviour since food prices are potential barriers towards sustainable and healthy food choices (Barosh et al. 2014; Thow et al. 2014).

## Challenges to achieving healthy and sustainable food systems

There are many challenges to achieving a global healthy and sustainable food system. A mass shift to healthy dietary preferences alone would require substantial policy reform (Hawkes et al., 2013). This section will discuss two specific barriers that are not traditionally considered in relation to healthy sustainable diets.

### Impact of climate change on agriculture, nutrition and health

A major challenge that can affect the sustainability of diets is environmental change, in particular climate change. Water scarcity, heat stress and levels of carbon–dioxide (CO_2_) and tropospheric ozone (O_3_) are forecasted to increase substantially (Collins et al. 2013; Kirtman et al. 2013). These stressors pose a significant threat to global agricultural production both in terms of harvestable yield and nutritional quality of crops, which could have a dramatic effect on food security, nutrition, and population health worldwide (Springmann et al. 2016; Myers et al. 2017; Whitmee et al. 2015).

There is general consensus that changes in rainfall and temperature will lead to significant reductions in the yields of staple crops (Challinor et al. 2014; Jerry et al. 2012; Lobell et al. 2011; Zhao et al. 2017). Impacts will be more profound in tropical than in temperate regions in the near term. Crop yields in temperate area are expected to increase during the first half of the 21st century, followed by yield reductions in the second half of the century, when temperatures are anticipated to exceed the optimal growing range of several crop groups (Knox et al. 2016).

It has been suggested that fruits and vegetables are particularly sensitive to temperature changes, for example due to their relative low “failure point temperatures” (Backlund et al. 2008). Furthermore, these crop groups are highly vulnerable to visible damage—predominantly caused by increased concentrations of ozone—which could substantially reduce their marketability (Peet and Wolf 2000). Environmental stressors have also been found to negatively affect the nutritional composition of crops: increased concentrations of CO_2_ have been identified to lower specific nutritional concentrations, notably zinc and iron concentrations of staple crops and fruits and vegetables (Myers et al. 2014; Scheelbeek et al. 2017). Reduced yields and lower nutritional quality of food crops increases the likelihood of food shortages and nutrition insecurity especially in low-income countries where the projected effects of environmental change are likely to be the most severe. Fruits and vegetables are of particular relevance for healthy and sustainable diets, given their unique composition of nutrients, their critical role in prevention of non-communicable disease and pre-mature mortality and their relatively lower resource input requirement (IHME 2017; Miller et al. 2017). With current worldwide per capita consumption of fruit and vegetables well below the minimum daily recommended level (FAOSTAT 2018), further reductions in fruit and vegetable availability and nutrient concentrations could pose threats to population health, particularly in areas with low dietary diversity. Furthermore, their reduced availability could complicate a suggested move towards more sustainable plant-based sources of protein—such as legumes—in future diets.

In the context of global efforts to promote healthy and sustainable food systems and meet the SDGs, it is critically important for policy planning and agenda setting to recognise the impacts and complex interactions of environmental stressors on agricultural production, nutrition and health (Tuomisto et al. 2017). Furthermore, it will be crucial to re-evaluate agro-ecological suitability of land, and its environmental conditions, to maintain yields and quality of food crops and safeguard global food security.

### Interaction between sustainable food systems, health, and industrial crop systems

Food production systems can interact strongly with industrial crop^3^ production systems. Considering the growing demand for bioenergy and other plant-based industrial products it is expected that the production of industrial crops will increase in the next decades (OECD/FAO 2017). This can cause a competition for land with food systems, with this competition, however, depending significantly by crop type, mode of production, environmental context and future use scenarios (e.g., Ahlgren and Di Lucia 2014; Dislich et al. 2017; Hertel et al. 2013; Gasparatos et al. 2018), At the same time, industrial crops such as sugarcane and oil palm are considered elements of non-healthy diets, so their increased production and consumption raise concern (FAOSTAT 2018).

This suggests that industrial crop systems can intersect in multiple ways with sustainable and healthy food systems, both synergistically and antagonistically. Promoting sustainable diets can either entail the sustainable production of some of the healthy industrial crops, such as cacao, and/or reduce human consumption of unhealthy ones, such as palm oil. Both approaches can have very important trade-offs that are particularly challenging to unravel. Understanding and harnessing these trade-offs is highly complicated and will increasingly become a major challenge in promoting healthy and sustainable food systems in the face of land competition and environmental change (see above), especially in developing countries as discussed below.

Reducing or changing the markets for industrial crop commodities in an effort to promote sustainable diets might have pronounced economic effects in some emerging and developing economics. For example, several developing and emerging economies have, specialised their agricultural systems to produce industrial crops. Malaysia/Indonesia are now the leading producers of oil palm, Brazil/India of sugarcane, and Ivory Coast/Ghana of cocoa, to mention just a few examples (FAOSTAT 2018). Sugarcane is one of the main foreign exchange earning sectors for Malawi and dominates the national economy in Swaziland and Mauritius (Chinangwa et al. 2017).

Apart from strong effects on the national economy, the production of industrial crops can have substantial local effects on livelihoods and food security. Mudombi et al. (2016) found that sugarcane plantation workers and smallholders in Malawi and Swaziland register significantly lower levels of multi-dimensional poverty compared to control groups (mainly subsistence farmers). Similar positive livelihood effects have been found for example for oil palm smallholders in Indonesia and cocoa producers in Ghana (Lee et al. 2014). When it comes to local food security, the effects of industrial crop production multiple drivers often manifest simultaneously in areas that food and industrial crop systems co-exist (Wiggins et al. 2015), which can influence food security in different ways (Dam Lam et al. 2017).

Unsustainable practices in industrial crop production can add further strains to the environment from agricultural systems (e.g., Dislich et al. 2017). For example, oil palm agriculture has been associated with substantial land use change in Indonesia and Malaysia, with knock-on effects on biodiversity loss, GHG emissions, and air pollution and water pollution (Carlson et al. 2012, Dislich et al. 2017; Obidzinski et al. 2012). However, some industrial crop systems can have, on some occasions, positive environmental outcomes. For example, Romeu-Dalmau et al. (2016) found that sugarcane plantations in Malawi and Swaziland sequester more carbon compared to previous land uses.^4^

The above are some of the strong linkages between food and industrial crop systems that can catalyse the emergence of important trade-offs when promoting sustainable and healthy food systems. They need to be considered thoroughly to minimize any negative trade-offs in developing countries that strongly depend on industrial crops for their national economy and livelihoods of local communities.

## Discussion

To sustainably feed the world’s growing population, reduce malnutrition and improve public health, major changes in food systems are required. Currently, there is a conducive international policy environment for the promotion of healthy and sustainable food systems. The SDGs generate new opportunities for creating and implementing cross-sectoral interlinked strategies and actions at both the global and local level that can facilitate further development towards healthy and sustainable diets. In particular, SDG2 (End hunger, achieve food security and improved nutrition and promote sustainable agriculture) and SDG3 (Ensure healthy lives and promote well-being for all at all ages) are closely interlinked. Studies of the interlinkages between the different SDG targets showed that these two SDGs are among the top four SDGs regarding the amount of interlinkages with other SDGs (ICSU 2107). Thus, interventions towards achieving one of these two SDGs may contribute meeting several other SDGs (ICSU 2107). Furthermore, overlapping with the SDGs is The UN Decade of Action on Nutrition 2016–2025 with the aim of eradicating malnutrition in all its forms in all countries, and with the first of its six focus areas being “Sustainable food systems for healthy diets and improved nutrition” (UNSCN 2016). In addition, meeting the Paris climate agreement will not be possible without a global transformation of the food system that includes rapid decarbonisation of the entire value chain, from food production to consumption (UNFCCC 2016).

Policies and other measures that are needed to increase the sustainability and nutritional values of diets differ depending on the local context. In general, emerging economies need to consider both the nutritional and sustainable aspects of rapidly shifting dietary patterns, whereas high-income countries need to focus on reducing the consumption of animal products in favour of plant-based diets. An increase in the consumption of animal products may, on the other hand, be needed to reduce malnutrition and ensure healthy development in children in some low-income contexts.

Health and sustainability often go hand in hand. Healthy food items have in general lower environmental impacts than food systems of unhealthy food. Tilman and Clark (2014) showed for example that red meat production has by far the highest land requirement and GHG emissions per gram of protein, than other systems. Sustainable agriculture has, in addition to positive impacts on the environment (e.g., IPSI 2018) also beneficial effects on health, for example through reduced exposure to harmful compounds such as pesticides. The concept of nature-based solutions and ecosystem services may be used within rural and urban farming systems to provide multiple benefits including direct and indirect health co-benefits (Lindgren and Elmqvist 2017; Takeuchi et al. 2016). Traditional integrated aquaculture–agriculture farming systems provide a range of multiple benefits. Cultivation of fish in rice paddies increases both the rice harvest and fish yields. The fishes act as biological pest controls by feeding on rice pests such as the golden snail (Halwart and Gupta 2004), thus contributing to both food security and household incomes. In addition, some fish species also eat mosquito larvae, which is of importance in areas where rice paddies are used as breeding sites by malaria-transmitting mosquitoes (Wu et al. 1991).

Aquaculture (plant and animals) is currently the fastest growing of all food sectors. In 2025, aquaculture is expected to provide 57% of all fish for human consumption (FAO 2016). Inland aquaculture will become increasingly important as both a source of nutrition and poverty alleviation in many rural areas in LICs. Since aquaculture is rapidly growing more research, monitoring of environmental impacts and development of sustainable production methods are urgently needed.

Innovations and food system technologies may help meet the raising demand for healthy and sustainable food. In cities, laboratory production of new food items, such as mycoprotein-based food, and new indoor techniques such as hydroponic farming could, in addition to outdoor urban farms and food gardens, contribute to local food production. However, the potential supply, consumer acceptability and overall impact of such systems are still not well understood.

Environmental impacts of industrial crop systems often reflect the management practices adopted, rather than the crops themselves (Gasparatos et al. 2018). Certification schemes have been proliferating in the past decades for different industrial crops such as sugarcane, oil palm and cocoa to mention some. Such market-driven approaches offer price premiums to producers to incentivise the adoption of environmentally and socially responsible practices during the production of industrial crops, as a means of enhancing their sustainability (e.g., von Geibler 2013). Perhaps efforts to promote healthy and sustainable diets, by curbing, for example, sugar intake, can be linked with the tighter enforcement of such certification standards (to reduce further environmental impacts) and provision of sustained premiums to producers to offset the livelihood costs of a declining demand.

It is important to reduce FLW along the supply chain. Improving technologies, infrastructure and energy use are examples of actions needed at the production, storage and transport levels, in particular in low- and middle-income countries. New initiatives and trends such as finding new markets for odd-shaped fruits and vegetables that are currently discarded are examples of actions partly based on emerging consumer awareness. To be effective behaviour change strategies for reducing food waste as well as changing dietary contents need to be adapted to the local context and to different consumer groups. Affordable solutions based on consumers’ disposable income are a requisite for successful consumption changes. Subsidies, taxes, and trade agreements throughout the supply chain are, thus, important tools to guide production and facilitate consumption of healthy, sustainable food.

## Conclusions

The global community is facing major challenges in the near future from increasing demand for food fuelled by global population growth and changing dietary habits, coupled with unsustainable food systems that threaten to cross planetary boundaries. Progress towards healthy sustainable diets for all can be accelerated through the international and local work undertaken to meet the Sustainable Development Goals. There is an urgent need to shift away from unhealthy food with high environmental impacts, towards plant-based diets with low amounts of animal source items that have been produced sustainably. Depending on the local context, both new and traditional sustainable food-producing practices can be implemented. However, when promoting sustainable diets, it is important to consider the intersection between food systems and industrial crop systems, as a series of complex trade-offs might emerge. The needs of vulnerable groups such as pastoralists in low-income countries who depend on their livestock for their livelihoods should be considered in developing sustainable food systems. Governance and means of control could be used to shift the choice of food items and the production, transport, storage and handling of the food supply chain towards healthy products and practices with less environmental impacts. Food loss and waste should be reduced along the supply chain, and consumer awareness increased about both food wastage and healthy sustainable diets. The emergence of new sustainable food items and production technologies could contribute to freeing land to be used for other purposes than food production (e.g., industrial crops and reforestation), as well as safe-guarding biodiversity in both terrestrial and marine ecosystems.

## Footnotes

1 For the purpose of this paper, we define food systems as the production, marketing, transformation and purchase of food, as well as the consumer practices, resources and institutions involved in these processes (Global Panel 2014).

2 A sustainable food system “is a food system that delivers food and nutrition security for all in such a way that the economic, social and environmental bases to generate food security and nutrition for future generations are not compromised” (UN 2015c, page 32).

3 For the purpose of this paper, industrial crops refer to crops not used for food (e.g., cotton and tobacco) or crops that have non-food uses but are also integral components of the food industry without being staple crops (e.g., oil palm, and sugarcane) (Wiggins et al., 2015). Industrial crops can have multiple non-food uses such as fibre, bioenergy, and other industrial products. Some industrial crops can be mono-functional, used, for example, only for fibre (cotton), recreation (tobacco) or energy (jatropha), while others such as sugarcane can be multi-functional, being used as food supplement (i.e., sugar), fuel (i.e., ethanol), and materials (i.e., bioplastics).

4 This is largely due to the prior degradation of surrounding forests for timber and fuelwood and the fact that other prevalent agricultural practices (e.g., rainfed maize production) can store substantially less carbon (Romeu-Dalmau et al. (2016)).
